# Decoding subcellular RNA localization one molecule at a time

**DOI:** 10.1186/s13059-025-03507-8

**Published:** 2025-03-03

**Authors:** Josep Biayna, Gabrijela Dumbović

**Affiliations:** 1 Goethe University Frankfurt, Center for Molecular Medicine, Institute for Cardiovascular Regeneration, Frankfurt, Germany; 2 Cardio-Pulmonary Institute (CPI), Goethe University, Frankfurt, Frankfurt, Germany; 3 German Center of Cardiovascular Research (DZHK), Partner Site Rhein/Main, Frankfurt, Germany

**Keywords:** RNA localization, RNA, lncRNA, Single molecule, RNA imaging

## Abstract

Eukaryotic cells are highly structured and composed of multiple membrane-bound and membraneless organelles. Subcellular RNA localization is a critical regulator of RNA function, influencing various biological processes. At any given moment, RNAs must accurately navigate the three-dimensional subcellular environment to ensure proper localization and function, governed by numerous factors, including splicing, RNA stability, modifications, and localizing sequences. Aberrant RNA localization can contribute to the development of numerous diseases. Here, we explore diverse RNA localization mechanisms and summarize advancements in methods for determining subcellular RNA localization, highlighting imaging techniques transforming our ability to study RNA dynamics at the single-molecule level.

## Introduction

The localization of an RNA within the cell is integral to its functionality. In eukaryotic cells, a significant portion of messenger RNAs (mRNAs) are subcellularly localized during development and in homeostasis [1, 2]. While mRNAs are typically enriched in the cytoplasm for translation (with exceptions), long noncoding RNAs (lncRNAs) are distributed throughout the cell, predominantly localizing to the nucleus. This diverse subcellular localization enables lncRNAs to be involved in nearly all aspects of gene expression regulation, including genome organization, nuclear architecture, transcription, RNA processing, translation, and mRNA turnover [3]. In recent years, remarkable technological advancements have revealed exciting concepts of RNA regulation, bringing spatiotemporal RNA regulation to the forefront. Importantly, disruptions in RNA localization can cause or contribute to disease. For instance, the expansion of CTG trinucleotide repeats in the 3′ untranslated region (UTR) of *DMPK* causes nuclear retention of its RNA, leading to myotonic dystrophy [4]. Similarly, mutations in RNA-binding proteins (RBPs) involved in RNA metabolism impair RNA localization in neurons, contributing to diseases such as amyotrophic lateral sclerosis (ALS), fragile X syndrome, and spinal muscular atrophy [5–8]. Therefore, understanding the principles governing RNA localization is of great importance. Yet, despite significant progress, much remains to be uncovered about the mechanisms underlying RNA localization, the specific transcripts influenced by these processes, and their overall impact.

Here, we summarize the mechanisms of subcellular RNA localization regulation and provide a deeper focus on the emerging role of intron retention (IR). We discuss how single-molecule RNA imaging techniques continue to shape our understanding of RNA localization, highlighting the critical roles that sophisticated mechanisms play in guiding RNA molecules to specific subcellular destinations. We use several well-characterized mRNAs and lncRNAs to illustrate how the interplay between splicing outcomes, RNA localization signals, RBPs, RNA stability/decay, and cellular architecture regulates spatiotemporal RNA availability, which can be dynamically modulated during the cell cycle and in response to stimuli or stress.

## Eukaryotic cells are highly structured

**Fig. 1:**
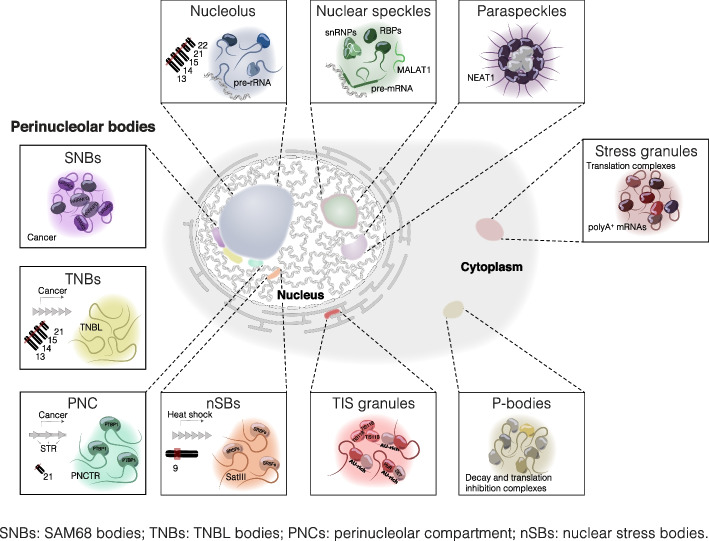
Eukaryotic cells contain diverse membraneless organelles. Examples of constitutively present and cancer- or stress-induced nuclear and cytoplasmic membraneless organelles, along with several of their characteristic RNAs and proteins. STR, short tandem repeats. Objects are not drawn to scale and do not represent all molecular constituents of the organelles

Eukaryotic cells are highly compartmentalized, with the nucleus and cytoplasm as principal compartments. This compartmentalization is facilitated by the nuclear envelope, which enables regulatory mechanisms specific to eukaryotic cells. For instance, in prokaryotes, mRNAs are translated concurrently with their transcription, while in eukaryotes, nuclear/cytoplasmic separation allows mRNAs to undergo processing before being exported [9]. The nucleus and the cytoplasm are further compartmentalized into specialized membrane-bound and membraneless organelles (Fig. 1). Membraneless organelles form without a surrounding membrane through a process known as biomolecular condensation, driven by multivalent protein–protein, RNA–protein, and/or RNA-RNA interactions, leading to phase separation of its components [10, 11]. The nucleus contains multiple membraneless structures, rich in proteins and noncoding RNAs, termed nuclear bodies, which provide compartments for diverse processes, including gene expression regulation, RNA processing, assembly of ribonucleoprotein (RNP) complexes, and the sequestration and modification of proteins [12, 13]. The best-described nuclear bodies include the nucleolus, perinucleolar bodies, Cajal bodies, nuclear speckles, paraspeckles, and polycomb bodies. Each nuclear body is characterized by its unique set or class of RNAs. For instance, Cajal bodies contain diverse proteins and RNAs crucial for the assembly, modification, and maturation of small nuclear and small nucleolar RNPs (snRNPs and snoRNPs) [14]. Paraspeckles assemble around the lncRNA *NEAT1*, which acts as a scaffold to recruit proteins through RNA–protein interactions. Within paraspeckles, *NEAT1* is highly structured: its 5′ and 3′ ends localize to the paraspeckle shell, while its central region is situated in the core [15]. Paraspeckles form at the *NEAT1* transcription site, typically near nuclear speckles, and have been implicated in nuclear RNA retention [16]. Nuclear speckles are dynamic structures enriched in pre-mRNA splicing regulators, including uridine-rich small nuclear RNA–protein complexes, serine/arginine-rich splicing factors (SRSFs), and lncRNA *MALAT1*, implicated in RNA splicing, mRNA quality control, and nuclear RNA retention [17–20]. Increased proximity of genes to nuclear speckles affects co-transcriptional splicing efficiency due to increased spliceosome concentrations and spliceosome binding [21]. Polycomb bodies are enriched in Polycomb group proteins and are considered gene repression centers, often near pericentromeric heterochromatin [22].

The nucleolus is the largest observable nuclear structure, forming around the nucleolar organizer regions on the short arms of acrocentric chromosomes and primarily serving as the site of ribosomal RNA transcription and biogenesis of pre-ribosomal particles [23]. The periphery of the nucleolus acts as a hub for transcriptionally inactive, repeat-rich regions, and pericentromeric heterochromatin [24]. Several structures can form adjacent to the nucleolus, often in association with stress or cancerous conditions, though their functions remain relatively unknown. These include the perinucleolar compartment (PNC), SAM68 nuclear body (SNB), and TNBL body (TNB) [25–29]. The PNC is enriched in RBPs and RNA polymerase III RNAs [25, 30, 31]. Its prevalence positively correlates with the metastatic capacity of various cancer types and has been proposed as a tumor diagnostic marker [32]. The formation of PNC is driven by the lncRNA *PNCTR,* which originates from ribosomal intragenic spacers on the short arms of acrocentric chromosomes and sequesters PTBP1, a regulator of alternative splicing [29]. SNBs contain members of signal transduction and activation of RNA (STAR) family of proteins, including SAM68, and primarily appear in cancer cells [26, 27]. TNBs, which form adjacent to SNBs, are driven by the primate-specific lncRNA *TNBL* transcribed from NBL2 macrosatellites in the pericentromeric regions of the short arms of acrocentric chromosomes in cancer [28]. Another primate-specific nuclear body is the nuclear stress body (nSB), which is often found near the nucleolus or nuclear lamina and forms in response to thermal and chemical stress due to the transcription of highly repetitive satellite III (HSATIII) lncRNAs and the aggregation of heat shock factor 1 (HSF1) [33–35]. nSBs act as a conditional platform for SRSF phosphorylation, promoting rapid adaptation of gene expression via IR following heat shock [36]. nSBs also sequester SAM68 and have been termed stress-induced SNBs [37]. A common theme among these perinucleolar compartments is their formation around tandem repeat-derived lncRNAs under stress or pathological conditions where these repeats are aberrantly expressed. They appear to be involved in splicing regulation, with the repetitive nature of these RNAs likely enhancing their ability to act as scaffolds.

The cytoplasm contains diverse compartments that spatiotemporally control post-transcriptional processes. These include membrane-bound organelles, such as the mitochondria, and those specialized in protein synthesis, sorting, and trafficking, such as the endoplasmic reticulum (ER) and Golgi apparatus [38, 39]. Cytosolic membraneless organelles include those that are constitutively present in cells, such as cytosolic processing bodies (P-bodies) and TIS granules, and those that emerge during stress, such as stress granules (SGs) [40–44]. TIS granules, enriched with TIS11B and membrane protein-encoding mRNAs with AU-rich elements in 3′ UTRs, intertwine with the rough ER and facilitate protein–protein interactions and co-translational assembly of protein complexes [41, 44]. P-bodies are formed by the co-assembly of translationally inactive mRNAs bound to various RBPs, including translation inhibition components and mRNA degradation components [45–48]. Similarly, SGs are assemblies of non-translating mRNAs and RBPs that form under stress when translation initiation is impaired, and they are thought to regulate stress response and post-transcriptional gene expression regulation [40].

## RNAs are dynamically localized

Subcellular RNA localization serves diverse functions in eukaryotic cells, including regulating the timing, location, and levels of protein synthesis, shaping cellular organization, and influencing RNA functions. Nearly 40 years ago, poly(A)^+^ RNA and β-actin mRNA were discovered to asymmetrically localize within ascidian eggs and embryos [49]. Many mRNAs were quickly found to have a nonrandom distribution within *Xenopus* and *Drosophila* oocytes, as well as differentiated somatic cells, and to colocalize with their encoded proteins [50–53]. This established intracellular mRNA transport as a potential mechanism to produce proteins at specific sites. With more sensitive and high-throughput approaches the number of localized mRNAs has drastically increased, revealing a global association between RNA localization, cellular architecture, and protein localization and function [1]. Screening 2314 transcripts expressed during *Drosophila* embryogenesis via high-throughput RNA FISH revealed that 71% exhibit developmentally-regulated subcellular distribution patterns [1]. Subcellularly, mRNAs display various patterns, including apicobasal gradients, tightly localized clusters, cell membrane associations, and enrichment at spindle poles, centrosomes, astral microtubules, and mitotic spindles. Some transcripts were predominantly nuclear, either localized perinuclearly or uniformly distributed.

Subcellular RNA localization is important for highly polarized cells, such as oocytes, migrating cells, and neurons. In many organisms, the establishment of the embryonic axis and cell fate depend on the asymmetric distribution of maternal RNAs within the developing oocyte [54]. For example, *oskar* mRNA, which encodes the posterior determinant Oskar protein in *Drosophila*, is actively transported to the posterior of the oocyte along a polarized microtubule network [55–57]. Importantly, *oskar* is kept translationally repressed before reaching the destination to prevent unlocalized Oskar protein production [56, 58–60]. In neurons, RNA localization to dendrites and axons enables local, often activity-dependent translation, supporting neuronal homeostasis, synaptic plasticity, and the autonomous function of neurites far from the cell body [52, 61–65]. Even in non-polarized cells, many mRNAs encoding non-membrane proteins are partitioned between the ER, TIS granules, and the cytosol [44]. TIS granule-enriched mRNAs typically encode low-abundance proteins, including transcription factors, whereas ER-enriched mRNAs encode large, highly-expressed proteins.

Transporting mRNAs instead of proteins has numerous potential benefits [66]. Localized translation lowers transport costs since multiple proteins can be generated from a single messenger, and it ensures proper protein function by spatially restricting their synthesis and preventing ectopic protein activity. Compartmentalized translation creates unique environments that facilitate protein interaction partner selection during synthesis, integration into complex structures, and response to signals. For example, most of the human mitochondrial proteins are encoded by the nuclear genome. After transcription, their mRNAs localize to the outer mitochondrial membrane for translation and the proteins are co-translationally imported into mitochondria [67, 68]. Localized translation of cytoskeleton regulators facilitates local cytoskeletal formation, cell polarization, and directed cell movement [66]. mRNA translation in TIS granules allows co-translational complex assembly [41, 44].

RNA localization can regulate translation levels. For instance, condition-dependent mitochondrial mRNA localization during respiratory conditions enhances protein synthesis in yeast [69]. The partitioning of mRNAs between the cytosol and the ER influences translation efficiency, with ER-localized mRNAs exhibiting higher ribosome loading [70]. Conversely, subcellular RNA compartmentalization can inhibit protein synthesis by sequestering the mRNA from the translation machinery. For example, during the antiviral response, the *mCAT2* gene produces *CTN*-*RNA* that accumulates in paraspeckles [71]. Its long 3′ UTR, containing inverted repeats subjected to adenosine-to-inosine editing, is cleaved upon stress, leading to export to the cytoplasm and translation. Nuclear RNA retention can also be regulated by IR, which occurs when one or more introns remain in an otherwise fully processed RNA, which will be discussed in the final section. Collectively, mRNA localization appears to be tightly regulated by the necessity for its encoded protein.

In contrast to mRNAs, whose localization is primarily dictated by the need for efficient protein synthesis, lncRNAs act directly through their RNA, making localization critical to their function. LncRNAs are remarkably versatile, found in nearly any subcellular compartment, and participate in a wide-array of cellular functions. They share similar sequence features with mRNAs and undergo comparable processing steps, including transcription by RNA polymerase II, 7-methyl guanosine capping, splicing, and polyadenylation [72–74]. However, lncRNAs differ in key aspects. They are generally less abundant, less efficiently spliced, and predominantly nuclear, often associated with chromatin at the transcription site and functioning in *cis* [74–76]. Some lncRNAs can be exported to the cytoplasm or localized in both compartments, where they engage in diverse non-coding functions [3, 77]. Moreover, certain lncRNAs undergo re-localization in response to cellular changes. For instance, *MALAT1* is exported to the cytoplasm of differentiating neurons, where it functions as a coding RNA to regulate synaptic activity [78]. Similarly, other lncRNAs with validated nuclear RNA-based functions were shown to produce proteins [79], highlighting the localization-mediated functional versatility of lncRNAs.

In summary, the eukaryotic cell is exceedingly complex, providing the cartography for diverse and distinct processes regulating chromatin architecture, transcription, RNA processing, translation, protein synthesis and degradation, complex assembly, cellular metabolism, and signal transduction. RNAs must navigate this landscape to localize correctly, which can in turn impact their post-transcriptional regulation and function.

## Methods to study subcellular RNA localization

Understanding the principles regulating RNA localization is crucial for elucidating RNA function. Therefore, techniques that identify subcellular RNA localization are essential for advancing RNA biology and have significantly improved in recent years, mainly due to advances in imaging technology.

Biochemical methods exploit the distinct physicochemical properties of cellular compartments, allowing their separation and RNA content analysis by RNA sequencing. These approaches enabled the mapping of RNA localization for a variety of subcellular compartments. For instance, fractionating the cell to cytoplasmic, nuclear soluble, and chromatin fractions has been widely used to determine differences in RNA localization between these compartments. This has been particularly valuable in lncRNA research, revealing that lncRNAs are mainly nuclear and chromatin-bound, and suggesting their roles in gene expression regulation through chromatin association or by acting in *trans* within the nucleoplasm or cytoplasm [36, 73, 80]. A recently developed method, LoRNA, determines cell-wide subcellular RNA localization by sorting subcellular components based on density, quantifying RNA abundance across density fractions, and leveraging localization-specific RNA correlation patterns to map the subcellular localization of the transcriptome [81]. Despite their advantages, biochemical methods have limitations: some compartments cannot be biochemically fractionated (such as the nuclear pore), they do not eliminate contaminants from other compartments, and they provide relative RNA quantity ratios.

Proximity labeling techniques have significantly enhanced the detection of RNA enrichment in specific subcellular compartments, particularly those not separated well with biochemical methods. APEX-RIP uses APEX (engineered ascorbate peroxidase)-catalyzed proximity biotinylation of endogenous proteins within a few nanometers, mild formaldehyde protein-RNA crosslinking, RNA immunoprecipitation (RIP), and sequencing [82]. APEX-RIP was applied to a variety of subcellular compartments and worked well for membrane-enclosed organelles, but performed poorly for non-enclosed regions, such as the cytosolic face of the ER. APEX-seq addressed these limitations by directly labeling RNA with APEX, eliminating the need for formaldehyde crosslinking [68]. APEX-seq generated a nanometer-resolution spatial map of endogenous RNA localization at nine subcellular locations. It revealed distinct paths of RNA localization to mitochondria, a radial organization of the nuclear transcriptome, and associations between the localization of mRNAs and the proteins they encode. PL-CLIP (proximity labeling-crosslinking immunoprecipitation) combines TurboID [83], a biotin ligase that biotinylates adjacent proteins without peroxide, thereby lowering toxicity, and UV-induced RNA–protein crosslinking [84]. Conjugating TurboID to the postsynaptic protein PSD95 enabled activation-dependent isolation and analysis of dendritic mRNAs and their associated proteins.

Proximity-labeling methods are restricted to analyzing a single subcellular compartment at a time, thereby limiting their ability to provide a comprehensive view of RNA localization. Therefore, multiple experiments must be combined to achieve a thorough understanding of RNA localization. Furthermore, they do not provide single-molecule resolution. To this end, single-molecule RNA fluorescence in situ hybridization (smRNA FISH) emerged as the leading technique for precise and reproducible imaging of RNA localization in single cells [85, 86]. Compared to traditional RNA FISH, which uses a longer DNA, cDNA, or RNA probe, smRNA FISH uses multiple (typically up to 48) single-fluorescently labeled oligonucleotides tiled across an RNA of interest [86–88]. smFISH benefits from a substantial signal-to-noise ratio since each oligonucleotide has one fluorescent label. Thus, their off-target hybridization lacks detectable fluorescent signal compared to tilling across their target. smRNA FISH can be combined with super-resolution microscopy techniques, including Stochastic Optical Reconstruction Microscopy (STORM) or Stimulated Emission Depletion (STED) microscopy [89–91]. This is particularly useful for studying the localization of RNAs accumulating at high density or for visualizing RNA–protein interactions [92, 93]. For example, STORM revealed between 50 and 100 *Xist* foci on the inactive X chromosome, contrary to the wide-coating mechanism suggested by conventional microscopy [93]. Similarly, STED revealed that TNBs are composed of dozens of *TNBL* molecules [28, 92]. However, smRNA FISH has several limitations. A minimum length of the RNA is required to hybridize a sufficient number of probes, limiting the pool of RNAs that can be visualized. It requires fixed cells, which is a significant drawback since RNA localization is inherently dynamic. Consequently, smRNA FISH is unable to provide insights into the temporal aspects of subcellular RNA distribution, leaving many questions about RNA localization dynamics unanswered. Furthermore, fixation and permeabilization alter the subcellular architecture [94].

To overcome these limitations, various live-cell RNA imaging approaches have been developed. The MS2-based system and its variations are the current gold standard for live-cell RNA imaging. The MS2 system involves inserting MS2 loops into the RNA of interest, which are then detected with the fluorescently tagged MS2 coat protein [95, 96]. Another widely used system, PP7, operates similarly by utilizing PP7 loops and its corresponding coat protein, enabling dual-color imaging and the development of tools such as single-molecule mRNA turnover biosensors [97, 98]. However, long MS2/PP7 arrays can interfere with local regulatory elements, RNA localization, or function. As an alternative, endogenous RNA dynamics can be imaged using molecular beacons (MBs), which consist of a target-complementary sequence, a fluorophore, and a quencher in a stem-loop structure, emitting fluorescence only when bound to their target [99]. MBs were used to visualize various localized mRNAs [100–102]. However, MBs have limitations, including off-target binding, degradation-induced fluorescence, targeting accessible sites, affecting RNA function/localization, sensitivity, and delivery. Other less intrusive methods are under development, such as live-cell RNA imaging techniques based on catalytically-inactive Cas13, which have been successfully applied to RNAs localized in granules or with repeated sequences, such as *NEAT1*, *SatIII*, *MUC4*, and *GCN4* [103]. However, achieving single-molecule resolution remains a challenge.

Due to the restricted availability of non-overlapping fluorophores, smRNA FISH can visualize only a limited number of RNAs simultaneously. To image multiple targets, high-throughput imaging techniques were developed, such as sequential RNA FISH (seqFISH) and multiplexed error-robust FISH (MERFISH) [104–108]. These approaches use optical barcoding through sequential rounds of readout probe hybridization, imaging, stripping, and re-probing. This is achieved by adapting the primary probe sequence, which is not fluorescently tagged, to include a target-complementary region and branches for binding of fluorescently tagged readout probes. They represent the best choice for high-throughput RNA mapping due to their unparalleled comprehensiveness and resolution. High-throughput imaging technologies can be coupled with DNA FISH and/or immunofluorescence, enabling the visualization of RNA and protein and/or nuclear architecture simultaneously [109, 110]. However, their adoption is technically demanding, requiring a sophisticated setup and expertise for effective incorporation. Another limitation is optical crowding and the requirement for non-overlapping signals from individual RNAs for accurate quantification, limiting RNAs that can be targeted. MERFISH addresses the density problem with sample expansion, which physically stretches the sample to increase the distance between RNA molecules [111], while seqFISH + detects fewer RNAs during each imaging round [112]. Alternative imaging-based subcellular RNA profiling techniques leverage in situ sequencing (ISS). Here, RNA is reverse transcribed, targeted by padlock probes that are subsequently amplified by a rolling circle amplification reaction and sequenced in situ [113]. Multiple variations of the ISS method have been introduced, including hybridization-based ISS (HybISS) and untargeted techniques such as fluorescence in situ sequencing (FISSEQ) and ExSeq (a combination of FISSEQ with expansion microscopy) [114–117]. These techniques offer the added advantage of detecting shorter RNAs. However, rolling circle amplification reduces single-molecule resolution, a key strength of MERFISH/seqFISH, and ISS-based techniques are equally technically demanding.

Although transcriptome-wide imaging approaches are efficient in capturing spatial RNA profiles, they lack the temporal resolution. Recently developed temporally resolved in situ sequencing and mapping (TEMPOMap) integrates metabolic RNA labeling and 3D in situ RNA sequencing to spatiotemporally track subcellular RNA kinetics [118]. TEMPOMap revealed differential regulation of RNA kinetics among functionally different genes. However, it is technically challenging to implement in routine laboratory work.

Overall, even though subcellular RNA dynamics are critical determinants of RNA function and cellular processes, their thorough characterization remains challenging. The method of choice will depend on the ultimate scope of the analysis. Integrating complementary techniques could provide a more comprehensive understanding of RNA localization and dynamics. smRNA FISH remains the gold standard due to its resolution, sensitivity, and unique ability to reveal detailed aspects of subcellular RNA localization. It shows whether RNA forms condensates/clusters, localizes in *cis*, or is widely distributed, as well as the number of molecules at each location. These observations are crucial for interpreting RNA function, especially for lncRNAs, which will be discussed next. Continued technological improvements are necessary to capture the complexity of RNA behavior within the cell and, consequently, to understand the regulatory networks governing cellular functions. The tremendous advancements in RNA imaging and tracking hold promise that soon we might be able to capture two key aspects of RNA biology, RNA localization, and dynamics, with a similar ease as we perform gene expression analyses.

## Understanding lncRNAs through imaging

Since lncRNAs exert their functions through the RNA, imaging techniques have been instrumental in elucidating lncRNA biology. They enable the visualization of RNA distribution throughout the cell cycle, determination of RNA localization relative to transcription and target sites, mapping of distribution patterns within the cell, and assessment of their splicing status. As an illustrative example, the exact mechanism of *Xist* in deactivating the female X chromosome was elucidated using RNA FISH, which demonstrated that *Xist* covers the inactive X chromosome on the same chromosome [119, 120]. RNA FISH has been applied to nearly all well-defined lncRNAs. Extensive smRNA imaging of 61 lncRNAs revealed a variety of localization patterns and provided new insights into lncRNA functions, including abundant focal nuclear localization; dispersed nuclear localization with or without foci; nuclear and cytoplasmic distribution; and exclusive cytoplasmic localization [77]. Visualization during the cell cycle revealed that most nuclear, chromatin-bound lncRNAs are released to the cytosol upon nuclear envelope breakdown and the onset of mitosis, suggesting they establish their role in chromatin de novo following cell division [28, 77]. Since tissues and cell cultures contain cells with heterogeneous expression profiles, smRNA FISH allows studying lncRNAs and their target genes at the single-cell level, providing indispensable insights compared to bulk assays. For instance, bulk RNA-seq indicated a positive correlation of expression between *bxd* lncRNA and its target *Ubx*, whereas smRNA FISH showed that their expression is mutually exclusive across cells, highlighting the repressive role of *bdx* [121].

RNA FISH enabled the discovery of a specific class of nuclear RNAs called architectural RNAs (arcRNAs), which define subnuclear compartments. For instance, *SATIII* lncRNAs were found to specifically accumulate in nSBs under stress conditions, *MALAT1* and *NEAT1* were found to accumulate in distinct nuclear regions tightly associated with nuclear speckles, and *TNBL* to form perinucleolar clusters in cancer cells [17, 28, 35]. Since then, numerous lncRNAs have been found to accumulate in nuclear compartments, which was crucial in defining the functions of many of these compartments [122].

smRNA FISH facilitates the characterization of lncRNA mechanisms by identifying both their site of origin and their final destination through co-staining against their intronic regions, which are present only at active transcription sites if the intron is efficiently spliced [123]. This allows to determine whether the RNA undergoes processing at the transcription site and transport of the mature transcript to its functional location in *trans*, or whether the RNA remains and functions in *cis*. Furthermore, targeting probes to both exon and intron regions enables the visualization of the splicing status of individual transcripts and their subcellular localization, with retained introns detected through exon–intron overlap outside transcription sites. This approach was instrumental in revealing that numerous mRNAs and lncRNAs are retained in the nucleus in an intron-retained (IR-) form. More recently, the distinction between mRNAs and lncRNAs has become increasingly blurred by the discoveries of lncRNAs with protein-coding potential [79, 124]. In this context, smRNA FISH has been particularly valuable in revealing lncRNAs with coding capacity, as demonstrated for lncRNA *MALAT1* [78].

In summary, subcellular lncRNA dynamics have an essential role in gene expression regulation. smRNA FISH has significantly advanced our understanding of how localization governs lncRNA functions, underscoring the importance of spatial regulation in RNA biology.

## RNA localization mechanisms

RNA localization is essential for its function, which necessitates identifying the sequence features that can predict RNA localization and function, similar to how protein function is inferred from its sequence. Despite significant progress, the complexities of RNA localization regulation have hindered the development of a unified model. RNA localization occurs through diverse mechanisms, including RNA–protein, RNA-RNA, and RNA–DNA interactions, often involving sequence-encoded localization signals or zip codes. Zip codes are predominantly found in the 3′ UTR, though not exclusively, and vary in length and complexity, ranging from short to long and from singular to multiple interacting sequences. Often, the secondary RNA structure consisting of stem-loops is more critical than the primary sequence for interactions with RBPs or other RNAs [125].

**Fig. 2:**
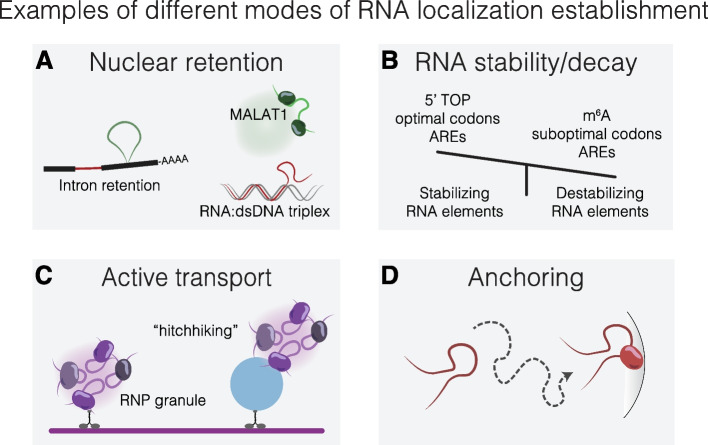
Examples of the distinct mechanisms by which subcellular RNA localization can be established. **A** RNA can be retained in the nucleus, for instance, by associating with nuclear bodies (e.g., nuclear speckles like *MALAT1*), retention of introns (exons in black, spliced intron in green, retained intron in red), or RNA:dsDNA triplex interactions. **B** Differential RNA stability and decay at various subcellular locations can influence RNA localization. This may be affected by stabilizing elements (e.g., 5′ terminal oligopyrimidine tracts (5′ TOP), optimal codons) or destabilizing elements (e.g., m^6^A modifications, suboptimal codons). For RNAs containing AU-rich elements (ARE), stability and decay depend on the associated RBPs. **C** RNA can be actively transported along cytoskeletal filaments by motor proteins, either independently or by “hitchhiking” on membrane-bound organelles such as lysosomes. **D** RNA localization can also be achieved through diffusion and subsequent local anchoring or entrapment, facilitated by interactions with specific cellular components

One of the first identified RNA zip codes was a 54-nucleotide sequence in the 3′ UTR of β-actin mRNA, initially discovered in fibroblasts [126]. Subsequent studies showed that this zip code is recognized by the RBP ZBP1, which orchestrates β-actin mRNA transport while maintaining it translationally inert. Upon reaching its destination, ZBP1 phosphorylation by Src kinase triggers its release, enabling β-actin synthesis and spatially-restricted actin filament formation [127]. Numerous studies have since identified RNA localization elements, including single or multiple stem-loops in *oskar*, *bicoid*, *gurken*, and *ASH1* mRNAs, among others [57, 128–133]. However, their systematic identification remains challenging due to the inherent complexity of RNA localization regulation, as discussed above, involving diverse processes, including active transport, transport inhibition, nuclear retention, diffusion, local anchoring, and localization-specific RNA stability or decay (Fig. 2) [125, 133].

Recent studies employed massively parallel reporter assays (MPRAs) using fragments (75–260-nt) tiled across the 3′ UTR of neurite-localized mRNAs for high-throughput identification of RNA localization elements [134–136]. These studies identified new RNA zip codes, including (AU)_n_ motifs, let-7 binding sites, and A/G-rich sequences. However, localization elements were found in a fraction of tested mRNAs, highlighting the complexity of RNA localization regulation and the limitations of MPRA in capturing longer or distributed elements. Differential RNA stability, influenced by *cis*-acting stabilizing or destabilizing elements that recruit *trans*-acting proteins, can play an important role in regulating RNA localization. For example, asymmetric RNA localization between neurites and stroma is driven in large part by differential stability, primarily due to the depletion of mRNA-destabilizing elements in mRNAs localized to neurites [137]. Several elements can regulate RNA stability. The 5′ terminal oligopyrimidine tracts in the 5′ UTR serve as stabilizing elements [138]. In contrast, post-transcriptional *N*^6^-methyladenosine modifications typically act as destabilizing elements and trigger RNA decay [139, 140]. AU-rich elements in 3′ UTRs can stabilize or destabilize RNA depending on the bound RBPs [141]. Codon optimality also affects RNA stability; transcripts with optimal codon sequences have faster translation elongation and longer half-lives, whereas those with suboptimal codons have slower translation rates and are more rapidly degraded [142–145]. Subcytoplasmic compartmentalization of mRNAs encoding non-membrane proteins into TIS granules, the ER, or the cytosol correlates with a combinatorial code based on total mRNA length, coding sequence exon length, and 3′ UTR-bound RBPs [44].

RNA localization can occur through passive diffusion and mRNA anchoring to specific subcellular sites [146, 147]. However, many RNAs rely on active transport, which is facilitated by zip code-interacting RBPs that associate with cytoskeletal motor proteins or adaptor proteins, enabling directed movement of RNA along cytoskeletal filaments to distal sites within the cell [55–57, 148–150]. Notably, mRNAs often travel packaged into RNP complexes or RNA granules, which act as dynamic hubs for RNA transport and regulation [147, 151–154]. Intriguingly, RNA granules can “hitch-hike” on membrane-bound organelles such as lysosomes, which are themselves coupled to motor proteins, for long-distance transport [155].

Although RNA–protein interactions are the most studied mechanism regulating RNA localization, intermolecular RNA-RNA interactions also play a significant role [148, 156]. For instance, protein-free RNA can self-assemble in vitro [10], and SGs have a bias toward longer RNAs, attributed to non-specific RNA-RNA interactions [157]. Specific interactions also contribute to RNA transport. Two well-described examples include *oskar* [158, 159] and *bicoid* mRNAs [160] where RNA granules, which are essential for mRNA transport, form via kissing-loop interaction that promotes dimerization or multimerization of RNAs. While the extent to which RNA-RNA interactions influence localization remains to be fully determined, it is hypothesized that they are common in areas with high local RNA concentrations, e.g., *NEAT1*-driven paraspeckle formation, and modulated by ribosome and RBP binding, underscoring the complexity of RNA localization regulation.

The predominant nuclear localization of lncRNAs has sparked significant interest in uncovering the factors that regulate their nuclear retention, yielding detailed mechanistic insights into specific lncRNAs. The lack of translation in most cases allows for a more flexible arrangement of localization signals. For instance, a pentamer sequence AGCCC was shown to drive the nuclear retention of the lncRNA *BORG* [161]. Two longer regions on opposite sides of *MALAT1* lncRNA were identified to regulate *MALAT1* nuclear retention and its association with nuclear speckles through interactions with nuclear speckle components [162]. The middle domain of *NEAT1* is essential for paraspeckle assembly by facilitating the recruitment of paraspeckle proteins [163]. RNA–DNA interactions, such as those seen in triple-helix forming lncRNAs in *cis* or in *trans*, also contribute to nuclear retention by anchoring RNAs to specific sites, thereby coupling RNA function with DNA loci [164, 165]. Despite the advances made with individual RNAs, no general features can explain the widespread nuclear retention observed in lncRNAs and certain mRNAs. Transposon-derived sequences further contribute to nuclear RNA retention [166]. Lubelsky and Ulitsky screened libraries of fragments tiled across 37 nuclear lncRNAs and mRNAs and identified a 42-nucleotide, Alu-derived, cytosine‐rich (C-rich) sequence driving nuclear RNA retention through interaction with HNRNPK, designated SIRLOIN (SINE-derived nuclear RNA localization) [167]. Similarly, Shukla and colleagues identified a C-rich motif over-represented in many nuclear-enriched regions using a MPRA on 38 lncRNAs [168]. In both studies, these sequences were sufficient to promote nuclear retention of otherwise cytoplasmic RNAs. However, such elements are absent from some highly nuclear lncRNAs, such as *XIST* or *NEAT1*, showing that nuclear RNA retention is influenced by more than one pathway.

Several machine learning and computational models have been developed to globally predict RNA localization from its sequence. deepLncRNA [169] and lncLocator [170] predict nuclear or cytoplasmic localization of lncRNAs, while RNATracker predicts the localization of mRNAs to the nucleus, cytosol, or membrane [171]. RNA‐GPS is based on a comprehensive subcellular RNA localization dataset achieved with APEX-seq [68, 172] and indicates that splicing and IR are strong predictors of subcellular RNA localization, as previously suggested [173] and further discussed in the following section.

## IR as a modulator of subcellular RNA localization

Splicing and mRNA export are functionally coupled. Splicing enhances the efficiency of mRNA export, and in some cases, splicing is essential for correct RNA localization in the cytoplasm [174, 175]. IR can regulate the transcriptome through multiple mechanisms, including RNA stability, premature stop codons, and subcellular localization. For a long time, IR was considered transcriptional noise, leading to RNA degradation [176]. However, multiple genome-wide and RNA-specific approaches have highlighted the relevance of IR in transcriptome regulation. A significant portion of mRNAs and lncRNAs have high retention of one or more introns, while other introns in the same transcript are efficiently spliced [76, 177–179]. Notably, lncRNAs show higher IR levels compared to mRNAs [74, 76, 177, 178, 180]. IR profiles vary between cell types, suggesting cell-type-specific functionalities, and can be modulated by intracellular or extracellular signals, and the cell cycle stage [76, 177, 178, 181, 182]. Boutz and colleagues analyzed IR in mouse embryonic stem cells (mESCs) and proposed two distinct classes of introns within processed transcripts [178]. Detained introns remain in the RNA in the nucleus until a splicing cue prompts their removal, enabling RNA export and translation. In contrast, retained introns are never spliced out. Transcripts with retained introns can be exported to the cytoplasm and translated or targeted for nonsense-mediated decay. However, distinguishing between transiently detained and stably retained introns is challenging. For simplicity, we will refer to introns present in processed transcripts as retained.

In mammals, IR is highly prevalent and can inactivate transcripts that are less needed or not required [177, 183]. This selective inactivation is essential for cellular homeostasis and for fine-tuning gene expression in response to developmental cues and environmental changes. Aberrant IR associates with various cancers, where it can contribute to transcriptome diversification and inactivation of tumor suppressor genes through premature stop codons [184, 185]. The assembly of nSBs promotes IR in a subset of transcripts during thermal stress recovery, thereby allowing for rapid adaptation of gene expression after heat shock [36]. This highlights the dual role of IR in both normal cellular functions and disease processes, underscoring its complexity and importance in gene expression regulation.

**Fig. 3:**
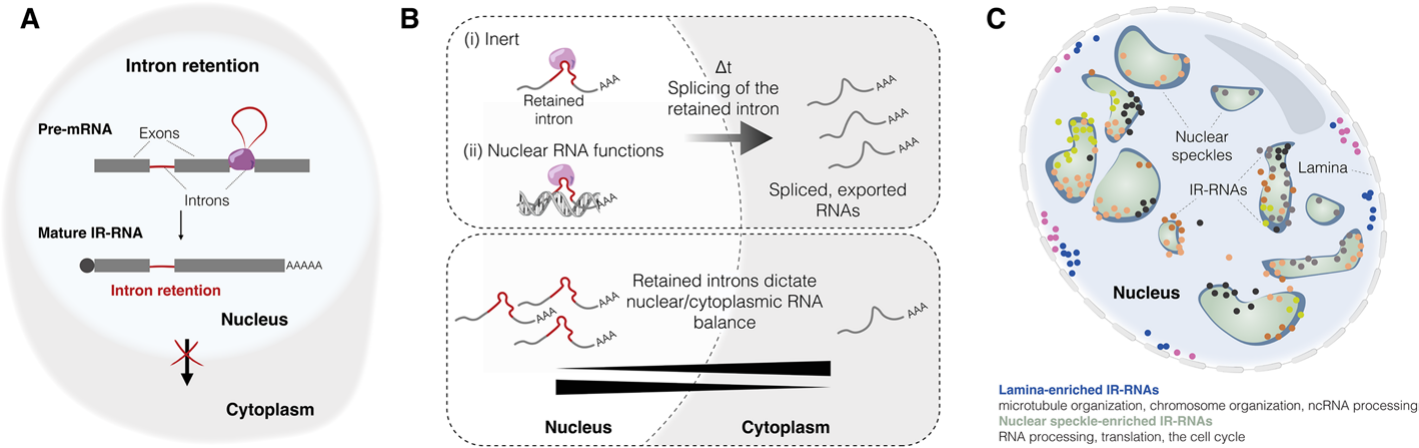
IR regulates subcellular RNA localization. **A** IR occurs when one or more introns remain in a fully processed and polyadenylated RNA, frequently resulting in nuclear retention of the RNA. **B** IR can regulate RNA function in various ways, such as keeping the RNA translationally inert in the nucleus or providing novel functional regions, among other mechanisms. The temporal regulation of IR and RNA export can affect RNA function and shape its nuclear/cytoplasmic balance. **C** IR-transcripts are associated with specific nuclear compartments [182]. For instance, IR-RNAs enriched at the nuclear lamina are linked to ncRNA processing, microtubule organization, and chromosome organization, whereas nuclear speckle-enriched IR-RNAs are involved in RNA processing, cell cycle regulation, and translation

The presence of retained introns has important functional implications for the RNA. Frequently, IR-transcripts are not exported to the cytoplasm, acting as a translational buffer and keeping mRNAs in a repressed but poised state [76, 177, 178, 186] (Fig. 3A, B). For instance, PTBP1-mediated IR in the 5′ UTR of transcriptional regulator *YY2* induces its translational suppression in mESCs, thereby regulating self-renewal and lineage commitment [187]. Transcripts encoding the SR protein kinase Clk1 accumulate in the nucleus with two unspliced introns, which are spliced out in response to heat or osmotic shock, and when Clk kinase activity is inhibited [188]. Inhibition of Clk activity affects IR in other transcripts, such as those that code for SR proteins or proteins involved in RNA processing [178]. In quiescent mouse muscle stem cells, high IR affects approximately 1200 transcripts involved in RNA splicing, translation, cell cycle, and lineage commitment. Among them is *Myod1*, a myogenic factor, whose IR keeps the RNA nuclear until Dek protein initiates intron removal, prompting quiescence exit [179]. *TERT* RNA, which encodes the catalytic subunit of the telomerase complex that elongates telomeres, has two retained introns and predominantly localizes in the nucleus [76, 189]. This indicates that RNA localization mechanisms regulate telomere homeostasis. These introns are spliced out during mitosis, suggesting that spliced *TERT* mRNA is inherited during cell division. Differential IR can regulate RNA localization and function in a species-specific manner, thereby influencing evolutionary changes in RNA function. For example, in human and murine ESCs, the lncRNA orthologs *hFAST* and *mFast* exhibit distinct localization and function. *hFAST* regulates Wnt signaling in the cytoplasm of hESCs, while *mFast*, influenced by the splicing inhibitor PPIE, remains nuclear and inactive in mESCs due to IR [181].

Within the nucleus, IR-RNAs associate with distinct compartments. Using APEX-seq, Barutcu and colleagues demonstrated that IR-transcripts predominantly associate with nuclear speckles and the nuclear lamina [182]. Notably, lamina and nuclear speckles are linked with functionally distinct IR-RNAs, partly attributed to intron features (Fig. 3C). However, the precise localization of IR-RNAs relative to nuclear speckles remains undetermined, limited by the resolution of APEX-seq.

Apart from sequestering the RNA in the nucleus and rendering it translationally inert, in some exciting cases, the long intronic sequences provide compartment-specific structural and binding regions that enable IR-RNAs to exert additional functionalities compared to their spliced counterparts (Fig. 3C). These additional properties include protein- and chromatin-binding regions, regulation of gene expression in *trans*, and the generation of nuclear bodies. For example, the lncRNA locus *Charme* produces an IR-isoform (*pCharme*) bound to chromatin that plays important roles in myogenesis by controlling the 3D proximity of myogenic domains [190]. On the other hand, the spliced, cytoplasmic isoform (*mCharme*) has no evident in vitro functional roles in myogenesis. *pCharme* retains intron 1, which facilitates the formation of nuclear condensates through interaction with PTBP1 and MATR3. Similarly, the evolutionarily conserved lncRNA *TUG1*, located in both the nucleus and the cytoplasm, has non-coding and potentially coding functions [76, 77, 191]. Nuclear *TUG1* retains two introns, whereas cytoplasmic *TUG1* is fully spliced, indicating that distinct isoforms from the same locus have different subcellular localizations and functions [76, 191].

While most IR-RNAs are nuclear, some localize to the cytoplasm and may interact with translation machinery [192–196]. Intriguingly, retained introns can regulate the localization of the IR-RNA to dendrites, mediated by Staufen2 [197, 198]. In ALS, an aberrant IR program results in a subset of cytoplasmically localized IR-RNAs that can bind ALS-associated proteins, contributing to a mislocalization-prone environment for these RBPs [199, 200].

In summary, IR is a widespread mechanism of gene expression regulation, with the potential to not only regulate translation but also to contribute to the diversification of RNA function and provide novel functions specific to distinct cellular compartments. Nevertheless, despite global and target-specific approaches, we lack a consensus understanding of which, why, or precisely when, transcripts are regulated by IR.

## Conclusions

RNA localization, a critical determinant of RNA functionality, is frequently overlooked. Advanced RNA imaging has greatly enhanced our understanding of spatiotemporal RNA dynamics at single-cell and single-molecule levels, setting a crucial foundation for lncRNA biology. However, observing RNA at a specific subcellular location does not necessarily imply a functional role, as it represents only a temporal snapshot. A major limitation is the scarcity of methodologies to visualize RNA's dynamic nature, coupled with the technical and expensive nature of existing high-throughput imaging approaches. These limitations leave gaps in understanding RNA localization regulation, timing, kinetics, and functions. The lack of known localization elements in most transcripts and the challenges in understanding their consequences hinder the discovery of the pathophysiological impacts of aberrant RNA localization. Integrating existing RNA localization data with accessible databases, such as the genome browser, would provide immediate access to subcellular RNA localization, and significantly advance the field.

It is important to note that distinct localization mechanisms apply to different groups of RNAs, each with specific roles and regulatory processes. In this context, emerging evidence suggests that IR-RNAs are uniquely regulated, potentially through distinct mechanisms or functions, with some examples discussed here. The complete spectrum of mechanisms regulating IR-RNAs, including the temporal, spatial, and functional aspects, remains to be elucidated. The retained intron may allow IR-RNAs to play non-coding roles in genome regulation, such as targeting genomic regions in *trans*, influencing higher-order genome architecture or scaffolding. IR-RNAs might collectively play a role in shaping nuclear architecture; their association with nuclear speckles may regulate speckle formation, shape, and/or size. The field of RNA localization is poised for exciting advancements, which will illuminate these still obscure aspects of gene expression regulation.

## Data Availability

No datasets were generated or analysed during the current study.
